# Development, characterization, and inter-laboratory validation of methylated human cell free DNA candidate reference materials

**DOI:** 10.1186/s13148-026-02156-3

**Published:** 2026-05-18

**Authors:** Zhiyong He, Yves Konigshofer, Russell Garlick, Jayanthi Ramprakash, Madhumita Ramesh, Eric Hall, Adam Corner, Michelle Clarissa, Jocelyn H. Wright, Victoria K. Cannon, Ming Yu, William M. Grady, Cecilia C. S. Yeung, Zachary Heimer, Zhili Wang, ShiPing Zou, Shidong Jia, Fang Liu, Giancarlo Bonora, Karol Bomsztyk, Daniel Mar, Kenneth D. Cole, Hua-Jun He

**Affiliations:** 1 National Institute of Standards and Technology, 100 Bureau Drive, MS 8312, Gaithersburg, MD 20899 USA; 2 LGC Clinical Diagnostics Inc., Gaithersburg, MD 20878 USA; 3 Bio-Rad Laboratories, 1000 Alfred Nobel Dr., Hercules, CA 94547 USA; 4 Translational Science and Therapeutics Division, Fred Hutchinson Cancer Center, Seattle, WA USA; 5 Clinical Research Division, Fred Hutchinson Cancer Center, Seattle, WA USA; 6 Department of Pathology, University of Washington, Seattle, WA USA; 7 Department of Medicine, University of Washington, Seattle, WA USA; 8 JBS Biosciences, 3805 Old Easton Rd, Doylestown, PA 10892 USA; 9 Pillar Biosciences, 9 Strathmore Rd, Natick, MA 01760 USA; 10 Predicine, 3555 Arden Rd., Hayward, CA 94545 USA; 11 UW Medicine South Lake Union, University of Washington, Seattle, WA 98109 USA

**Keywords:** Cell free DNA, DNA methylation, Reference material, Characterization, Interlaboratory study, Cancer detection, Epigenetics

## Abstract

**Background:**

Aberrant DNA methylation biomarkers have demonstrated potential for early cancer detection, multicancer detection, and determining the tissue of origin. Due to their stability, frequency, and accessibility in bodily fluids, circulating cell-free DNA (cfDNA) methylation is a promising biomarker in liquid biopsy. A reliable and quantifiable analysis of cfDNA methylation status is critical to its application. However, there are current challenges and a lack of consensus on measurement methods. To address this, we developed two candidate methylated cfDNA reference materials (RMs).

**Methods:**

The National Institute of Standards and Technology (NIST) RM consists of five components, formulated by mixing in vitro methylated cfDNA simulant at fractions of 0%, 5%, 25%, 50%, and 100% with native-state cfDNA simulant derived from the GM24385 cell line. The LGC Clinical Diagnostics (LGC) RM consists of two components: non-methylated cfDNA simulant derived from GM24385 genomic DNA and whole genome amplification and methylated cfDNA produced by in vitro methylation of amplified material. The candidate RMs were characterized, and the methylation status of three targets was confirmed by droplet digital PCR (ddPCR) assays. To test the utility of these RMs, six laboratories participated in an interlaboratory study, each using their own lab-developed assays and methods, which included methylation-specific qPCR, nanoplate digital PCR (dPCR), ddPCR, matrix methylated DNA immunoprecipitation-based assays, and whole-genome bisulfite sequencing.

**Results:**

The interlaboratory study results showed that the designed percentage of methylation was well correlated with the observed values across all participating labs, and good reproducibility was found for each individual method. However, slightly different methylation proportions associated with assay-specific biases were observed.

**Conclusions:**

This study clearly demonstrates the value of candidate RMs as standards for evaluating assay performance, as well as for increasing confidence in reporting cfDNA methylation status for clinical applications.

## Introduction

In mammalian cells, adding a methyl group to the position 5 of the cytosine (C) by DNA methyltransferases (DNMT) restricts the access of the transcription factors to the methylated promoter region, thereby regulating gene expression [1]. The methylation reactions mainly occur on the cytosine upstream of guanine (G) in the DNA double-helix, known as CpG dinucleotide. DNA methylation is one of the mechanisms cells use to regulate gene expression. DNA methylation can be affected by many factors, including endogenous biological processes such as differentiation [2–4] and disease pathology [5–7], as well as exogenous environment exposures such as tobacco exposure and alcohol consumption [8, 9]. These dynamic changes of DNA methylation can therefore be used as biomarkers for disease detection [10–12], tissue source identification and age estimation for forensic sciences [13–15].

DNA methylation is the most studied epigenetic modification in humans. It was also the first epigenetic abnormality identified in cancers and has been used as a cancer biomarker [16]. The cancer genome is characterized by aberrant DNA methylation, including genome-wide hypomethylation and site-specific hypermethylation [17]. The alterations of global DNA methylations are believed to contribute to cancer initiation [18]. In combination with tissue specific DNA methylation patterns, DNA methylation measurements can not only detect the abnormal methylation patterns of cancer but also distinguish the tissue source of the cancer [19]. Methylation-based multi-cancer detection has been reported recently [20].

Circulating cell-free DNA (cfDNA) consists of small DNA fragments found in body fluids such as blood, urine, and saliva [21]. The discovery of cfDNA has led to noninvasive prenatal testing [22] and identification of actionable tumor mutations for cancer therapy [23]. DNA methylation is a relatively stable alteration that can be detected from cfDNA in liquid biopsy with minimal or non-invasive methods. This provides the advantage of detecting cancer at early stage [24] and possibly diagnosing cancer. However, the advantage depends on specificity of the method and accurate cfDNA methylation measurement. Currently, the most popular DNA methylation measurement methods are based on cytosine conversion, where an unmethylated cytosine is converted into uracil while the methylated cytosine remains unchanged [25, 26]. These converted residues will be read as thymine, as determined by PCR or sequencing analysis using dU-tolerant polymerases. Recently, cytosine conversion can also be achieved with an enzymatic approach [27]. However, the challenge is that the measurements can vary significantly from experiment to experiment and from lab to lab [28]. For example, bisulfite sequencing progressively degrades DNA but failing to run the reaction long enough causes unmethylated cytosines to remain as cytosines.

An unmet need is that a set of well-characterized reference materials that can be used for all assay developers and end users. These reference materials can help address the analytical validity of methylation measurement and discordance of the cfDNA methylation measurements between the laboratories. Methylated cfDNA reference materials can increase the confidence and reliability of the measurements in liquid biopsy. Commercially available methylated DNA standards exist, such as those from Qiagen and Zymo Research. However, they are not provided in cfDNA format. Their genomic sequences are not well characterized. It is uncertain whether the consent has been obtained to make these genomic sequences public available. In this study, we generated candidate methylated cfDNA reference materials derived from the well-characterized genomic DNA of a Genome-in-a-Bottle (GIAB) human cell line GM24385 (HG002) to address this unmet need. Their utility and performance were subsequently evaluated in a pilot inter-laboratory study, demonstrating that these reference materials can be applied across multiple platforms and laboratories.

## Materials and methods

### cfDNA simulant preparation

Due to the low abundance of human cfDNA in plasma, it is not possible to obtain sufficient cfDNA for large scale methylated donor-derived cfDNA candidate reference materials. Alternatively, we fragmented Genome-in-a-Bottle cell line GM24385 (HG002) genomic DNA (gDNA) [29–31] and selected the DNA fragments with relevant sizes of cfDNA (50–300 bp, average 168 bp) to make cfDNA simulant. GM24385 gDNA (130 µL) was sonicated in Covaris microtube (Covaris, Cat# 520045) on a Covaris Sonicator S2 (Covaris, Woburn, MA), at an intensity of 5, duty cycle of 10% and cycle/burst of 200 for 4 cycles of 48 s. The fragmented gDNA was then diluted in Tris-EDTA (TE) buffer (Thermo Fisher Cat# AM9849) to the final concentration of 80 ng/µL. The diluted fragmented gDNA was aliquoted 200 µL in each tube and size selection (50–300 bp) was performed using AMPure XP Reagent (Backman Coulter, Cat# A63880) following the manufacturer’s protocol. One hundred and eighty microliters of AMPure XP Reagent (10:9 ratio) was added to each tube, and mixed. The supernatant from previous step was transferred to a clean tube and further added 722 µL of AMPure beads (10:19 ratio). The size selected cfDNA was analyzed for the size distribution by Bioanalyzer. LGC Clinical Diagnostics (SeraCare) candidate reference materials were generated with proprietary methods but were also based on GM24385 gDNA that used progressive rounds of amplification to reduce overall cytosine methylation to close to zero.

### In vitro methylation

Size selected cfDNA simulant was methylated in vitro by using M. SssI CpG Methyltransferase (New England Biolabs, Ipswich, MA, Cat# M0226). In each reaction, 2 µg of cfDNA simulant was treated with CpG Methyltransferase in a 100-µL reaction at 37 °C for 1.5 h. After in vitro methylation, the cfDNA simulant was purified and stored at -20 °C until use. Based on the published studies, *CCNA1* (Cyclin A1), *VIM* (Vimentin), and *ELV* (Ena/VASP-like) are frequently found to be hypermethylated in specific cancer types and have been validated as reliable cancer biomarkers [11, 32, 33]. We developed three targeted assays, designated CCNA1, VIM1, and EVL1, each specific to one of the genes. The primer and probe sequences used in these assays are detailed in a manuscript submitted elsewhere. Using these three NIST-developed assays, we quantified the percentage of methylated DNA for each target gene. Droplet digital PCR assays for human genome DNA fragment S2 and gene *RPL32* were used as reference genes for DNA quantification [33], methylation insensitive assay C-less-C1 was previously published [34]. All primers and probes were ordered from Thermo Fisher Scientific.

### Cytosine conversion

To measure the percentage of methylated cfDNA simulant, the cfDNA simulant was treated with bisulfite conversion kits (EZ DNA methylation-Direct kit, Zymo Research, Cat# D5020 and EpiJET Bisulfite Conversion Kit, Thermo Fisher Scientific, Cat# K1641) by following the manufacturer’s instructions. The bisulfite conversion kits were blinded as Kit A and Kit B in the Results section. The converted DNA was either analyzed via ddPCR immediately or stored at -80 °C till use.

### Candidate reference material sample formulation

After confirmation that nearly 100% of cfDNA simulant was methylated, the methylated cfDNA simulant was mixed with native status cfDNA simulant at the percentages of 0% (all native status cfDNA simulant), 5%, 25%, 50% and 100% (all methylated cfDNA simulant). The native status gDNA CpG methylation across the genome was characterized previously [35]. The mixed cfDNA simulant was then aliquoted 10 µL/tube (20 ng/µL) in Sarstedt tubes and stored at − 20 °C. The LGC cfDNA simulants were designed so that the 0% sample contained near completely unmethylated DNA across the genome (relative to the GM24385 baseline), while the 100% sample represented the same DNA but fully CpG methylated.

### Digital droplet PCR (ddPCR)

The percentage of cfDNA simulant methylation, cfDNA simulant homogeneity, and stability were examined by digital droplet PCR (ddPCR). The cytosine converted cfDNA simulant samples were mixed with ddPCR Supermix for Probes (No dUTP) (Bio-Rad, Cat# 1863024) and primer/probe set (final concentration of 900 nM for primers and 250 nM for probe. The reaction mixture was then subject to Bio-Rad manual Droplet Generator to generate the droplets according to the manufacturer’s instructions. The endpoint PCR was performed using the following protocol: 1 cycle of 95 ˚C for 10 min; 60 cycles of 94 ˚C for 30 s, 60 ˚C for 1 min; then 98 ˚C for 10 min. The positive and negative droplets were counted by QX200 Droplet Reader, and the data was analyzed using QX Manager Software 2.1 (Bio-Rad). Unconverted cfDNA simulant was used as control for recovery rate calculation.

### Stability and homogeneity analysis.

The stability of the methylated cfDNA candidate reference materials was analyzed by randomly selecting one set of the samples and examining the percentage of methylated cfDNA and the copy number of cfDNA detected for serial time points, from 1 month to 30 months at storage condition of -20 ˚C. The detected DNA copy number was determined by using C-less-C1 assay [34]. The percentage of the methylated cfDNA simulant was determined by using 3 assays (CCNA1, VIM1, and EVL1) and calculated as methylated DNA copies divided by total DNA copies (methylated + unmethylated DNA copies). The homogeneity of the candidate reference materials was assayed by testing the percentage of methylated cfDNA simulant and detected copy numbers of 5 randomly selected sets of the samples (Set number 21, 28, 59 72, and 80).

#### Interlaboratory study

Participating laboratories were recruited through an open invitation posted at NIST website and National Cancer Institute (NCI) Early Detection Research Network (EDRN) contacts. Each participating laboratory received a set of the candidate reference materials. The participant laboratories performed the cfDNA methylation measurements using individual lab developed assays and methods.

### Participant laboratories materials and methods

Participant Lab 1: The methylation status was tested via ddPCR using a set of 5 ddPCR assays designed for methylation sites (*CCNA*1, *CGI*3, *WIF*1, *HOXA*9, and *NPY*) that also contain recognition sites for *Aci*I, a methylation-sensitive restriction enzyme (MSRE). Each assay was tested in duplex with an assay for a control gene, *RPP30*, to correct for variations in sample addition. Each reaction contained 4.95 ng of test cfDNA in all template-containing wells. Reaction mixes for ddPCR were formulated with ddPCR Supermix for Probes (No dUTP), both with and without MSRE, then partitioned with an Automated Droplet Generator (Bio-Rad Laboratories, Cat# 1864101). Endpoint ddPCR was run on a C1000 Touch Thermal Cycler with 96-Deep Well Reaction Module (Bio-Rad Laboratories, Cat# 1851197) using the following protocol: 37 °C for 45 min, 95 °C for 10 min, 45 cycles consisting of 94 °C for 30 s and 55 °C for 60 s, 98 °C for 10 min, and 4 °C for 30 min. The reactions were analyzed with a QX600 Droplet Reader (Bio-Rad Laboratories, Cat# 12013328) and QX Manager analysis software (Bio-Rad Laboratories, Cat# 12018108). The levels of the methylated target sites were reported as percentage of internal control, which was detected copy concentration of target divided by copy concentration of internal control then multiplied by 100%. The results were from two independent operators to ensure the reproducibility of the test.

Participant Lab 2: As part of the Early Detection Research Network, the Biomarker Developmental Laboratories and Biomarker Reference Laboratories at Fred Hutchinson Cancer Center developed a predictive assay based on a multiplexed panel of methylation-sensitive biomarkers for early detection of esophageal adenocarcinoma (EAC) and high-grade dysplasia (HGD) using the QIAcuity digital PCR (dPCR) system. This assay contains three methylation sensitive primers sets (assay B, D and M) and one methylation-insensitive assay, *C-less-C1*, to use as an internal control to normalize for total amplifiable DNA in the reaction and was optimized to be run with the low input DNA amounts typically obtained in esophageal brushing samples. DNA was quantified with the Qubit Flex Fluorometer 1X dsDNA assay kit (Cat# Q33230), then was bisulfite converted using the Zymo Research EZ DNA Methylation Kit (Cat# D5002), normalized to the appropriate concentration, and run in duplicate at 4 ng/well on a QIAcuity One dPCR system (Qiagen, Cat# 911021) with Qiagen methylation commercial control added, Epitect 100% Methylated and Epitect 100% Un-Methylated DNA (Cat# 59695). Results were analyzed using the QIAcuity Software Suite. Analysis of the five blinded NIST cfDNA samples revealed differing levels of percent relative methylation at our three test loci. The mean values obtained had a linear relationship with the percent methylated DNA for each test sample reported by NIST. Reproducibility studies performed included inter-day, inter-operator, inter-run, and intra-operator. The results passed with % CV lower than 35%.

Participant Lab 3: For each of the NIST samples, 123 µL TE (Tris 10 mM, EDTA 0.1 mM, pH 8.0) was added to bring total volume to 133 µL. 20 µL (30 ng) was used for bisulfite conversion, and each sample was run in triplicates. Bisulfite conversion reagent, instrument and protocol was used according to the manufacturer (JBS Science, Cat# 08878). Bisulfited treated sample was eluted in 50 µL TE and 4 µL was used in a duplex qPCR assay in duplicate. The duplex qPCR assay target methylated *GSTP1* (HEX label) and methylated *RASSF1A* (FAM label) for Urine DNA hepatocellular carcinoma screening. Duplex assays were done according to assay kit supplier (JBS Science, Cat # qPCR-019). The results were from two independent operators to ensure the reproducibility.

Participant Lab 4: Moderately and severely formalin compromised DNAs (Horizon Discovery), and both methylated and unmethylated human standards (Zymo Research Corp.) were converted using either the Zymo EZ DNA Methylation-Lightning Kit or the Qiagen Epitect Fast Bisulfite Kit. Primers were designed with small amplicons ranging from 80 to 100 bp, and with gene compatibility in one primer pool. Each conversion reaction had 40 ng of cfDNA input, and each GS-PCR run had 15 ng of cfDNA input. Post bisulfite treatment, an amplicon based NGS Library preparation protocol consists of two rounds of PCR and subsequent DNA purifications were used to prepare libraries to be sequenced on Illumina sequencing platform. DNA methylation levels from 0 to 100% NIST samples, LGC samples were tested in duplicate for all 5 assays (BRCA1, BRCA2, RAD51C, XRCC3-1, and XRCC3-2). Libraries were sequenced on Illumina MiSeq and NextSeq machines, and sequencing data was analyzed through Bismark and PiVAT^®^ (Pillar Biosciences’ Variant Analysis Toolkit). The oncoReveal 4 Gene Methylation LBx Panel utilizes 12 distinct amplicons to investigate the methylation levels of CpG sites within key transcription regulation regions of *BRCA1*, *BRCA2*,* RAD51C*, and *XRCC3*, including promotor, UTR, and intronic sequences. These 12 pairs of primers were designed to amplify both methylated and unmethylated DNA after bisulfite conversion and generate amplicons diverse enough for NGS analysis with uniform coverage. Sequenced reads were analyzed using Pillar Biosciences proprietary PiVAT software, version 2023.1, with default settings.

Participant Lab 5: Whole-genome DNA methylation data were generated using the PredicineEPIC™ assay with cfDNA input amounts as low as 1 ng [36]. For this study, 15 ng of NIST cfDNA methylation reference materials were processed using proprietary PredicineEPIC™ library construction and bisulfite-free methylation chemistry. The resulting methylation-treated libraries were sequenced to ~ 30× whole-genome depth on the Illumina NovaSeq X Plus platform using 2 × 150 bp paired-end reads. Paired-end reads were processed using the Predicine DeepSea NGS analysis pipeline. Briefly, the pipeline performs adapter trimming, barcode checking, and error correction prior to alignment. Cleaned FASTQ files were aligned to the human reference genome (GRCh37) using BWA. Consensus BAM files were generated by collapsing paired-end reads originating from the same cfDNA fragment through UMI-aware deduplication. CpG-site methylation states and sequence variants were subsequently called directly from the consensus BAM. For each uniquely aligned DNA fragment, the fragment-level β value (fraction of methylated CpG sites) was calculated for fragments covering ≥ 4 CpG sites. CpG-level methylation was then computed as the average of fragment-level β values across all fragments covering that CpG. NIST 0%, 5%, 25%, 50%, and 100% methylation standards were differentiated by their mean CpG-level fragment-derived methylation values using genomic regions that exhibited strong contrast between 0% NIST replicates (average β < 2.5%) and 100% NIST replicates (average β > 90%) and that passed QC criteria. Finally, sample-level methylation values were normalized for differences in methylated-cytosine conversion efficiency to generate the final genome-wide methylation estimates for each sample.

Participant Lab 6: CfDNA simulants received from NIST lab was boiled and rapidly placed on ice to generate ssDNA. 96-well microplate-based Matrix methylated DNA immunoprecipitation (Matrix-MeDIP) of ssDNA was done as previously described where 5mC antibody was attached to wall wells via Protein A [37, 38]. Twelve and half nanogram of DNA was used for each reaction. Well walls were blocked with 5% BSA. PIXUL fragmented ssDNA was used as input. After washes, immunocaptured ssDNA was recovered by incubation with Proteinase K (200ng/µL) in 100 µL/well of elution buffer (55 ˚C for 45 min, 95 ˚C for 10 min, then 4 ˚C). MeDIP DNA was used in qPCR using indicated primers to the *MGMT* gene [38].

## Results

### Size distribution and copy concentration of the candidate reference materials

**Fig. 1:**
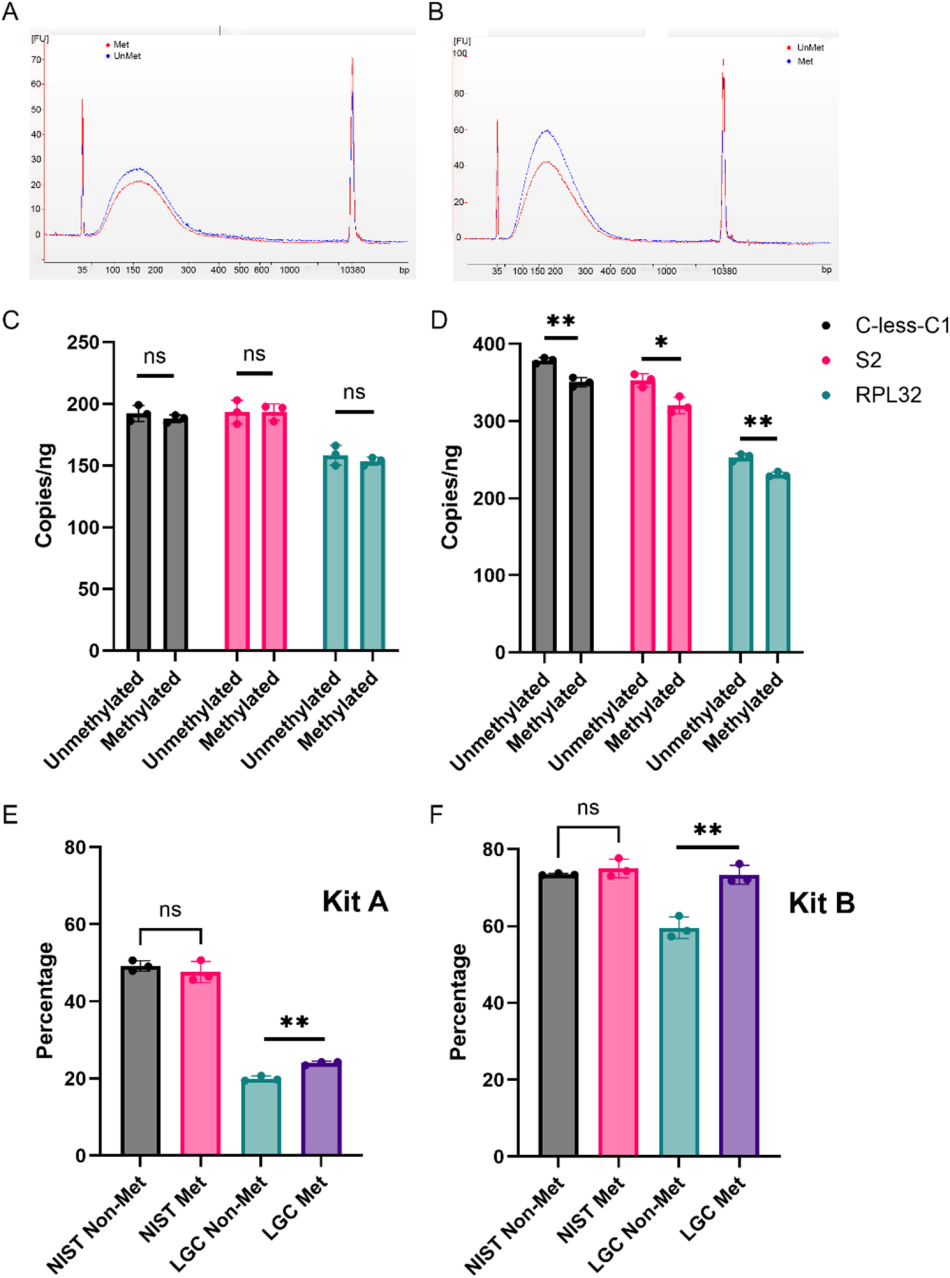
Size distribution, gene copy number and bisulfite conversion recovery rate of methylation and unmethylated candidate reference materials. A, Size distribution of NIST methylated and non-methylated candidate reference materials. B, size distribution of LGC methylated and non-methylated candidate reference materials. C, Detected copy numbers of reference genes in each nanogram of NIST candidate reference cfDNA. D, Detected copy numbers of reference genes in each nanogram of LGC candidate reference cfDNA. E, The recovery rate of methylated and non-methylated candidate reference materials after bisulfite conversion was determined by assay C-less-C1 with bisulfite conversion Kit (**A**). F, The recovery rate of methylated and non-methylated candidate reference materials after bisulfite conversion was determined by assay C-less-C1 with bisulfite conversion Kit (**B**) *, denotes *P* < 0.05, **, denotes *P* < 0.01. All data are presented as mean ± SD

We first tested whether there is bias of size distribution between the unmethylated and methylated cfDNA simulant. The size distribution was analyzed by Bioanalyzer. As shown in Fig. 1A and B, the size distribution of both NIST and LGC candidate reference materials were from 50 bp to 350 bp, and average size was 168 bp (Fig. 1A, B). This data indicated that non-methylated and in vitro methylated cfDNA simulant had similar size distributions.

We further investigated whether the copy concentrations of certain DNA targets were biased between the unmethylated and methylated samples. We utilized three ddPCR assays (C-less-C1, S2, and RPL32) to determine copy concentration of each sample. For the NIST samples, the unmethylated and methylated samples showed no statistical difference (Fig. 1C). However, the methylated sample from LGC showed 7–9% lower copy number than unmethylated samples for all 3 assays (Fig. 1D).

We also compared the recovery rate of the unmethylated and methylated samples after bisulfite conversion reaction. We performed the bisulfite conversion using 2 different kits (Kit A and Kit B hereafter). The recovery rate was calculated as the ratio of the C-less-C1 concentration detected in converted sample to that in sample before conversion reaction. Figure 1E and F showed that there was no recovery rate difference between the NIST unmethylated and methylated samples performed on Kit A and Kit B respectively. However, the LGC methylated sample showed higher recovery rate than the unmethylated sample (Fig. 1E, F).

### Methylation level of the candidate reference materials

**Fig. 2:**
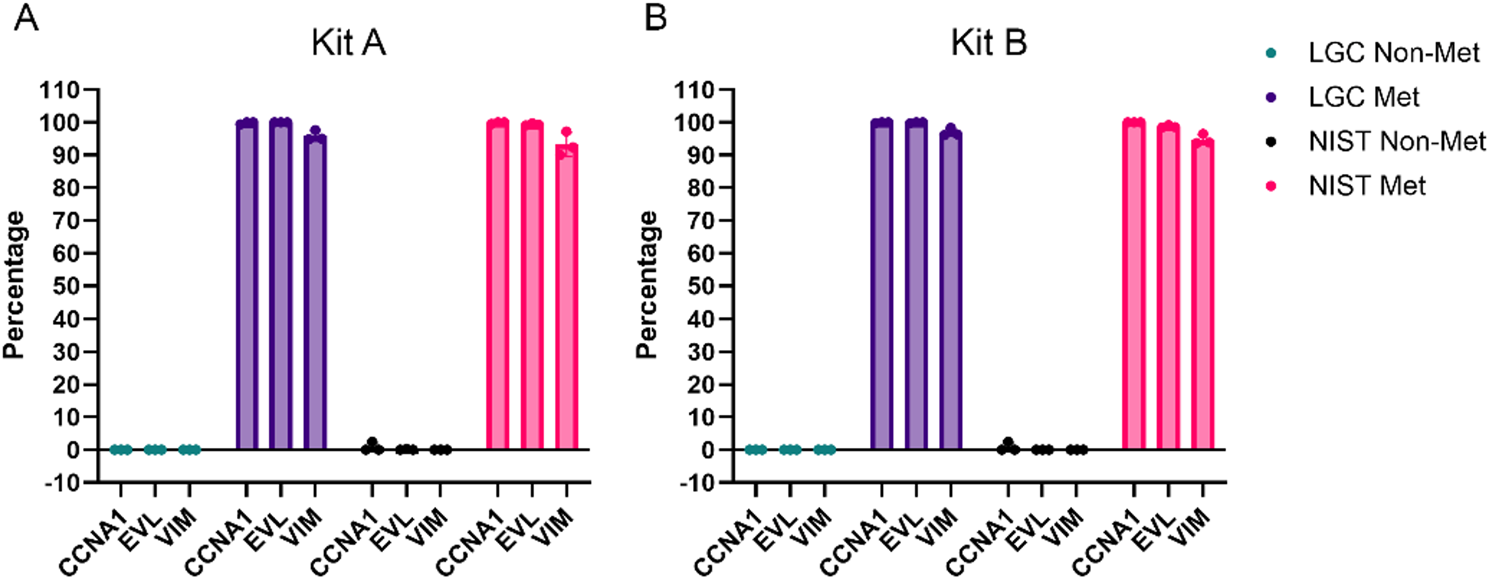
Methylation levels of 3 cancer biomarkers in methylated and non-methylated candidate reference materials. A, Methylation levels of 3 cancer biomarkers in methylated and non-methylated candidate reference materials detected by Kit (**A**). B, Methylation levels of 3 cancer biomarkers in methylated and non-methylated candidate reference materials detected by Kit (**B**) . All data presented are mean ± SD

**Fig. 3:**
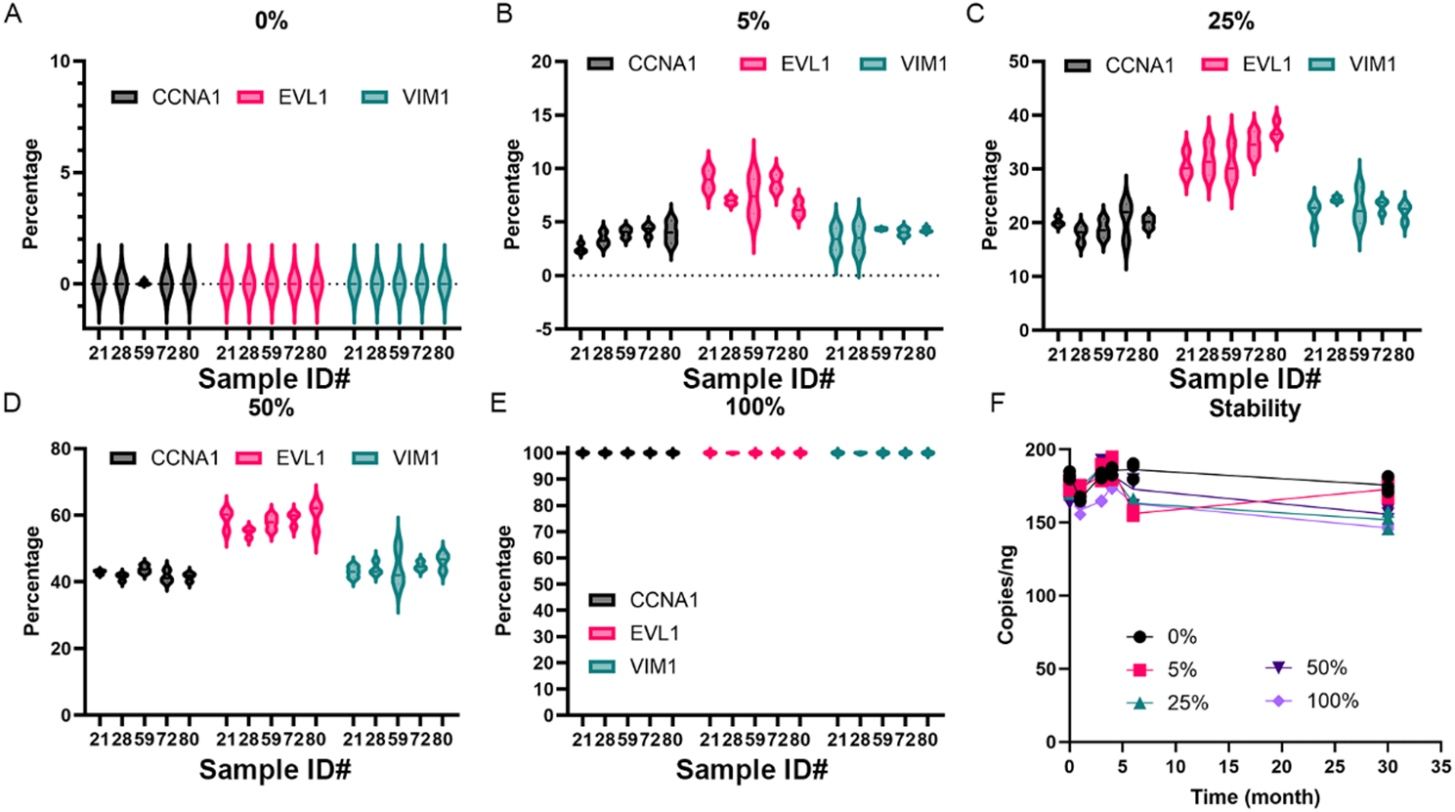
Homogeneity and stability of NIST candidate cfDNA methylation reference materials determined by ddPCR with Assay C-less-C1. A, Homogeneity of 0% methylated sample. B, Homogeneity of 5% methylated sample. C, Homogeneity of 25% methylated sample. D, Homogeneity of 50% methylated sample. E, Homogeneity of 100% methylated sample. F, Stability of NIST candidate cfDNA methylation reference materials up to 30 months stored at -20 °C determined by ddPCR assay C-less-C1. All data presented are mean ± SD

**Fig. 4:**
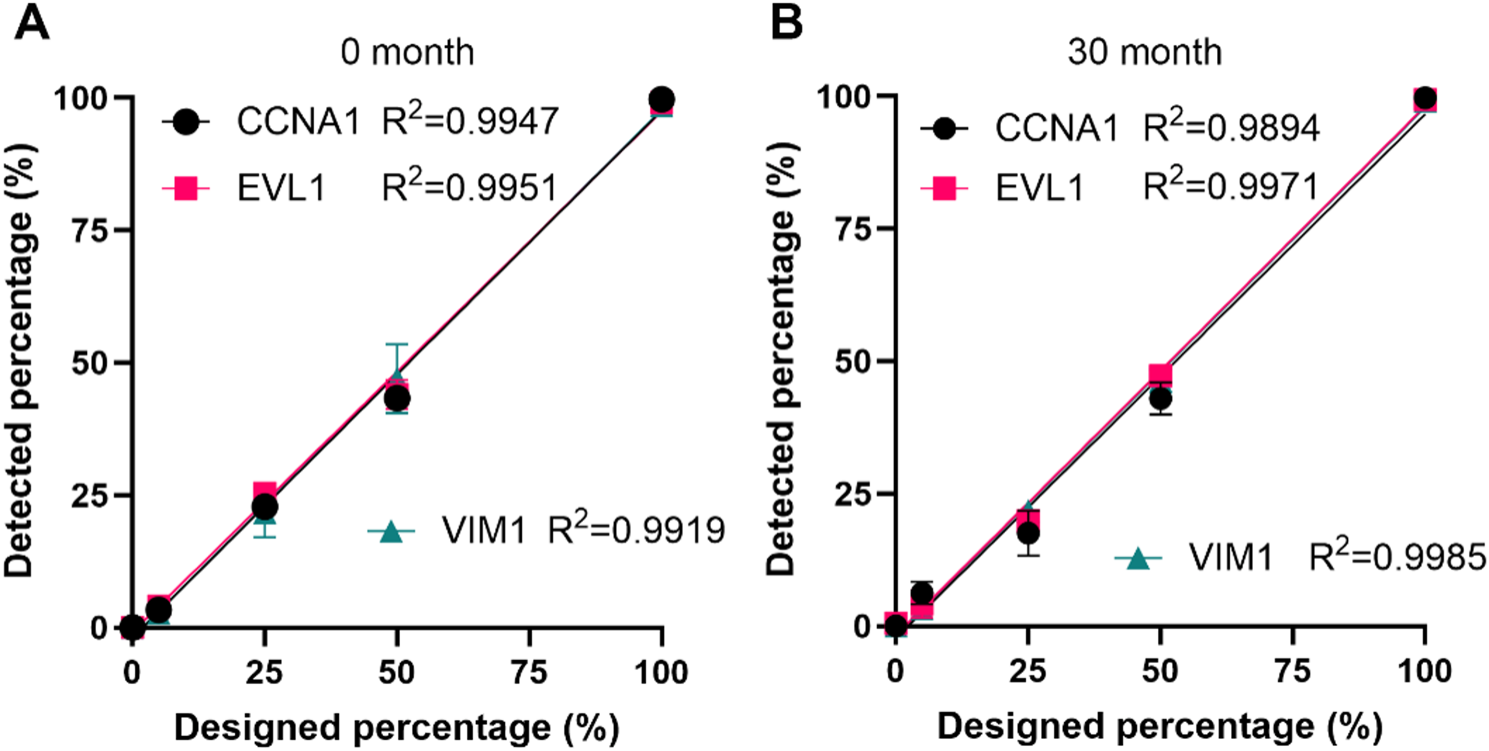
Correlation of detected and designed percentage of methylated cfDNA. A, Correlation of detected and designed percentage of methylated cfDNA measured with 3 cancer biomarkers at 0-month time point. B, Correlation of detected and designed percentage of methylated cfDNA measured by 3 cancer biomarkers at 30-month time point. All data presented are mean ± SD

We measured the percentage of methylated DNA with 3 assays, i.e. CCNA1, EVL1, and VIM1. NIST unmethylated candidate reference materials showed minimal background methylation for CCNA1 and 0% methylation for EVL1 and VIM1 (Fig. 2A, B). LGC unmethylated samples showed 0% methylation for all 3 assays (Fig. 2A, B). Both NIST and LGC methylated candidate reference materials were nearly 100% methylated for CCNA1 (99.85% ± 0.26% and 99.82% ± 0.31%, mean ± SD) and EVL1 (99.35% ± 0.30% and 100% ± 0.00%. The percentage of VIM1 methylation varied across experiments, ranging from 93.24% ± 3.68% to 99.97% ± 0.11% in the NIST sample (Figs. 2A, 3E and 4) and the percentage of VIM1 methylation in one experiment was 95.84% ± 1.55% LGC methylated sample (Fig. 2B).

### Homogeneity and stability of NIST candidate reference material

As mentioned in the Materials and Methods section, NIST candidate reference materials were formulated for 0%, 5%, 25%, 50%, and 100% methylated cfDNA. One set of NIST materials contained 5 individual samples. We produced 100 sets of candidate reference materials in this initial batch. The candidate reference materials were stored at -20 ˚C. To test the homogeneity of the candidate reference materials, we randomly selected 5 sets (set number 21, 28, 59, 72, and 80) and tested the percentage of methylated DNA using 3 assays (CCNA1, EVL1, and VIM1). Figure 3A-E showed that all the samples were homogeneous without any significant difference in mean among the samples (*p* > 0.05, one-way Anova test, *n* = 5).

To test the stability of the NIST candidate reference materials, the copy number of the cfDNA simulant was analyzed using the C-less-C1 assay at different time points. As shown in Fig. 3F, over the 0–30 month period, ddPCR analysis revealed no obvious trend of DNA degradation.

### Detected and designed percentage of methylated DNA correlation

We further analyzed the detected and designed percentage of methylated cfDNA correlation of the NIST candidate reference materials using 3 assays at 0- and 30-month time points. As shown in Fig. 4A, all 3 assays produced correlation curves close to the ideal line at 0-month time point. The R^2^ for CCNA1, EVL1, and VIM1 assay were 0.9947, 0.9951, and 0.9919 respectively, and the slopes were 0.995, 0.983, and 0.996 respectively. At the 30-month point, the R^2^ for CCNA1, EVL1, and VIM1 assay were 0.9931, 0.9979, and 0.9986 respectively, and the slopes were 0.958, 0.975, and 0.973 respectively. This data indicated that the correlation between the detected and designed percentage of methylated cfDNA support the use of this material as a reference material.

### Inter-laboratory study

Each inter-laboratory study participant received one set of NIST candidate reference materials and one set of LGC candidate reference (non-methylated and in vitro methylated cfDNA simulant). The participants performed the methylation analysis using lab developed assays. The platforms used by participants included ddPCR, nanoplate digital PCR, next generation sequencing, methylated DNA immunoprecipitation, and quantitative real-time PCR. The final report format included percentage of methylated cfDNA simulant, copy concentration, percentage of internal control, copies of methylated cfDNA simulant per nanogram of cfDNA simulant input, and fraction of input.

**Fig. 5:**
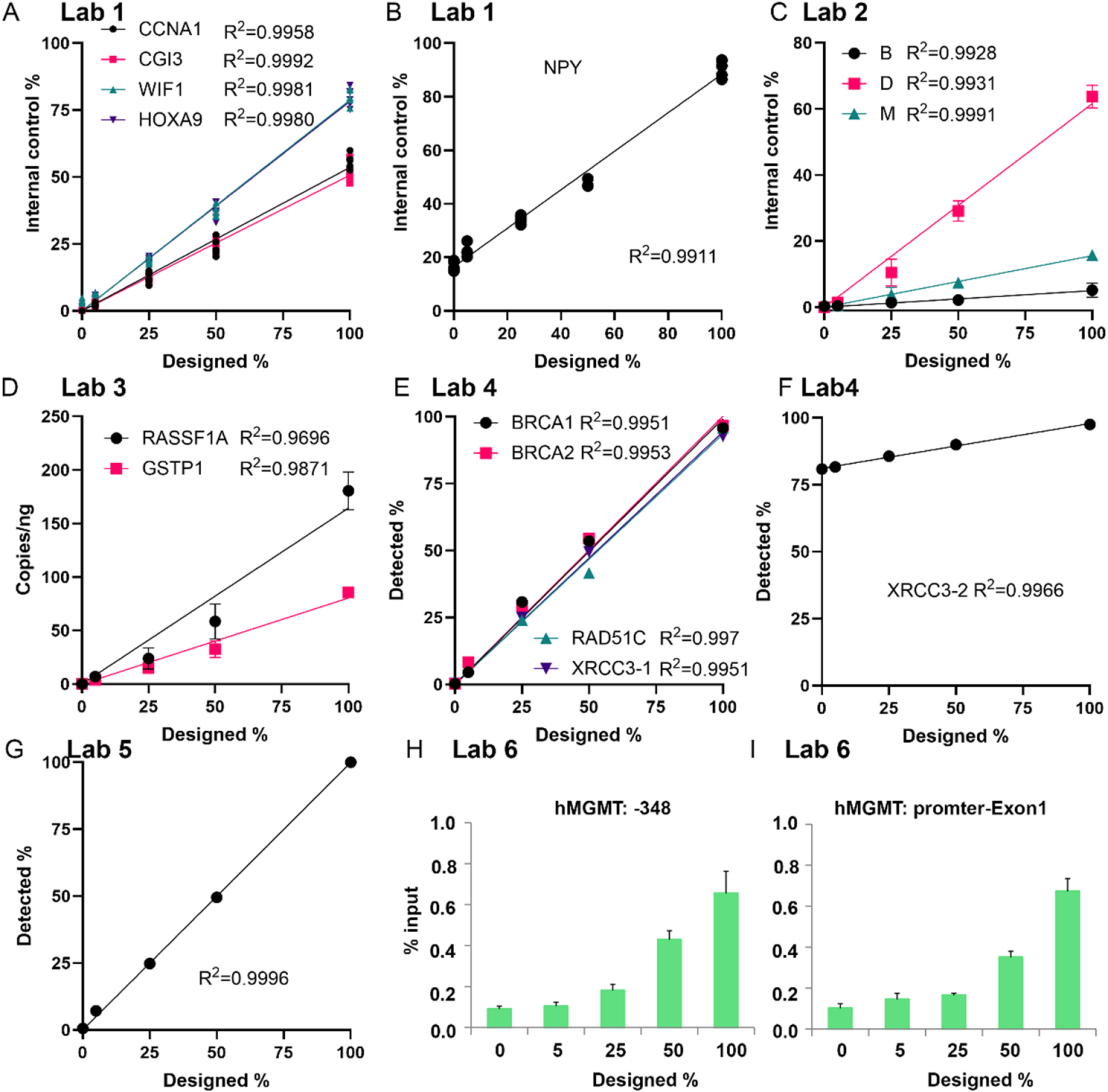
Test results for NIST candidate reference materials from participating labs. A, Correlation of detected percentage of methylated cfDNA relative to internal control and designed percentage of methylated cfDNA for 4 genes with minimal baseline methylation by Participating Lab (1). B, Correlation of detected percent of methylated cfDNA relative to internal control and designed percentage of methylated cfDNA for NPY with baseline methylation by Participating Lab (1). C, Correlation of detected percentage of methylated cfDNA relative to internal control and designed percentage of methylated cfDNA for 3 assays (Assay B, D, and M) with minimal baseline methylation by Participating Lab (2). D, Correlation of detected copy number of methylated cfDNA per nanogram of DNA and designed percentage of methylated cfDNA for 2 genes with minimal baseline methylation (*RASSF1A* and *GSTP1*) by Participating Lab (3). E, Correlation of detected and designed percentage of methylated cfDNA for 4 genes (*BRCA1*, *BRCA2*, *RAD51C*, and *XRCC3-1*) with minimal baseline methylation by Participating Lab (4). F, Correlation of detected and designed percentage of methylated cfDNA for *CRCC3-2* with high baseline methylation by Participating Lab (4). G, Correlation of detected percentage of cfDNA methylation and designed percentage of methylated cfDNA by Participating Lab (5). H, I, Correlation of detected percentage of input cfDNA and designed percentage of methylated cfDNA for 2 regions of *hMGMT* gene by Participating Lab (6). All data presented are mean ± SD

Participant Lab 1 performed 5 assays using ddPCR. Figure 5A showed the correlation of the designed percentage and the detected percentage of the internal control (*RPP30*) for four assays (copy concentration of detected methylated target/copy concentration of internal control %). These four assay targets had minimal background methylation in native cfDNA simulant (0% sample). All R^2^ values were greater than 0.995, indicating the correlation between the designed percentage and the detected percentage of the internal control were nearly totally aligned. Lab 1 also included an assay (NPY) target with background methylation in the 0% methylation sample. In the native state of cfDNA simulant, methylated *NPY* was approximately 17% normalized to the internal control (Fig. 5B). The correlation between the designed percentage and the detected percentage of the internal control was also nearly totally aligned (R^2^ = 0.999).

**Fig. 6:**
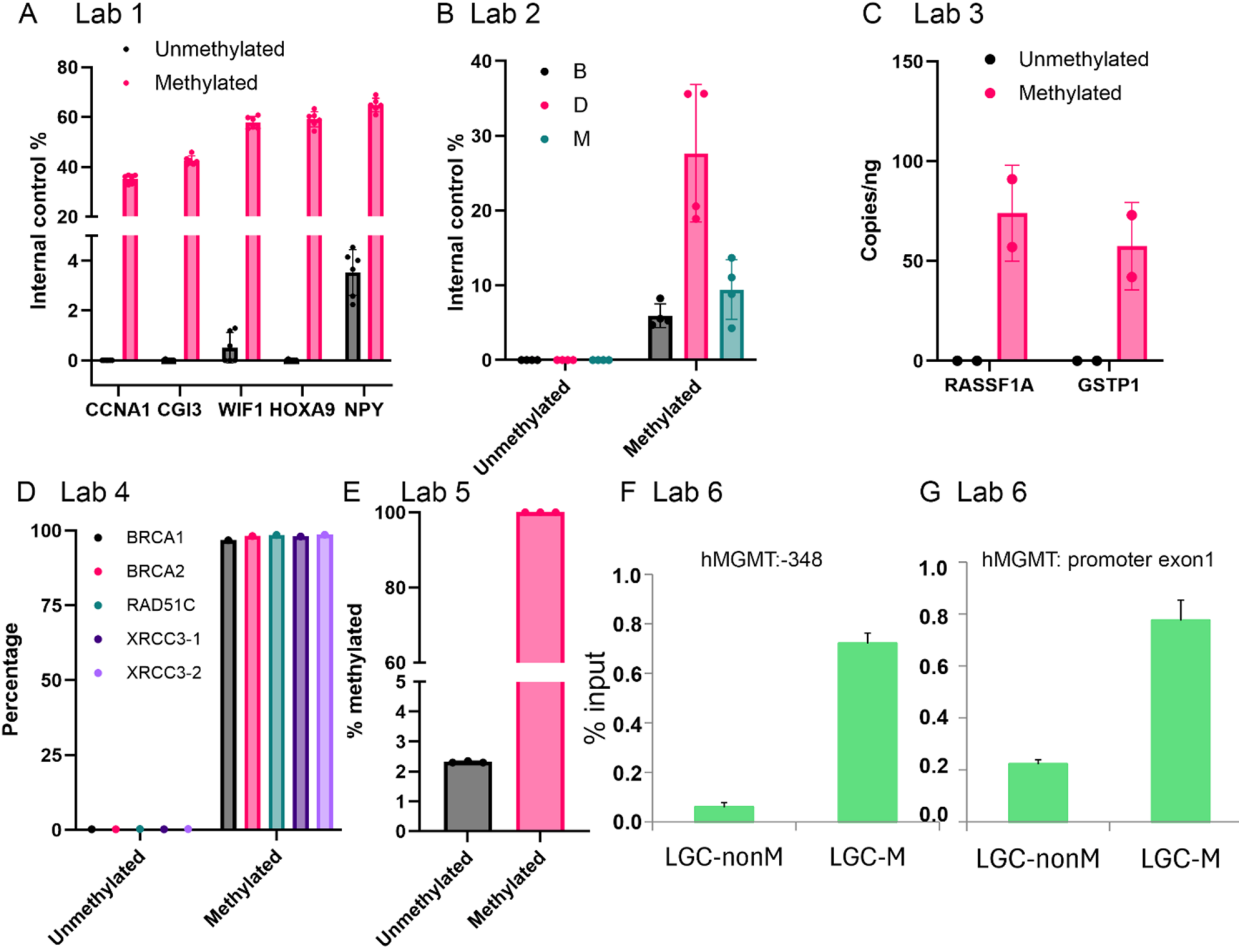
Test results for LGC candidate reference materials from participant labs. A, Detected percentage of methylated cfDNA relative to internal control for methylated and non-methylated cfDNA materials for 5 assays by Participating Lab (1). B, Detected percentage of methylated cfDNA relative to internal control ( Assay C-less-C1) for methylated and non-methylated cfDNA materials for 3 assays (Assay B, D, and M) by Participating Lab (2). C, Detected copy number of methylated cfDNA per nanogram of cfDNA for methylated and non-methylated cfDNA materials for 2 assays by Participating Lab (3). D, Detected percentage of methylated cfDNA for methylated and non-methylated cfDNA materials for 5 assays by Participating Lab (4). E, Detected percentage of methylated cfDNA for methylated and non-methylated cfDNA for 1 assay by Participating Lab (5). F and G, Detected percentage of methylated cfDNA relative to input cfDNA for methylated and non-methylated cfDNA materials for 2 assays by Participating Lab (6). All data presented are mean ± SD

Participant Lab 2 performed nanoplate-based dPCR using a multiplex assay with three methylation sensitive loci, assays B, D, and M, and one internal control locus (Assay C-less-C1). The correlation between the designed percentage and the detected percentage of the internal control (Assay C-less-C1) for all 3 methylation sensitive loci are shown in Fig. 5C, with all R^2^ > 0.99. The percent relative methylation (%RM) differed for all 3 loci in the multiplex similarly for the NIST standards and LGC controls shown in Figs. 5 and 6 and is consistent to differences between the individual loci with other samples tested by this laboratory.

Participant Lab 3 performed two assays with Real-Time qPCR (Fig. 5D). The reported readout was copies/ng. The correlation curves were shown in Fig. 5D, and the R^2^ values for *RASSF1A* and *GSTP1* were 0.97 and 0.987 respectively (Fig. 5D).

The report format from Participant Lab 4 was the detected percentage of methylated cfDNA. Figure 5E showed the correlation curves of the designed percentage and the detected percentage for 4 assays. The R^2^ values for these 4 assays were all greater than 0.995. The slopes for each assay were: 0.993 for *BRCA1*, 1.002 for *BRCA2*, 0.935 for *RAD51C*, and 0.944 for *XRCC3*-1. Participant Lab 4 also performed an assay (*XRCC3*-2) with high background methylation (80.87% methylation) in the 0% methylation sample (Fig. 5F). The R^2^ value was 0.9966 for this correlation (Fig. 5F).

Participant Lab 5 reported the detected percentage of methylated cfDNA. The correlation between the detected and designed percentage was shown in Fig. 5F, and the R^2^ value for this correction was 0.9996 and the slope of the curve was 0.999 (Fig. 5G).

Participant Lab 6 used methylated DNA immunoprecipitation (MeDIP) method. The reported readout was the percent of input. Figure 5H and I show that within the CpG island of the *hMGMT* gene, methylation at position − 348 and in Exon 1 above 25% is well correlated with the designed methylated cfDNA percentage.

For LGC candidate reference materials, most of the assays performed in this inter-laboratory study detected minimal background methylated cfDNA simulant in the non-methylated sample (Fig. 6A, B, C, D by Participant Lab 1, 2, 3, and 4). Background methylation was detected by assays NPY (Fig. 6A, Participant Lab 1), MeDIP (Fig. 6F and G, Participant Lab 6), and Participant Lab 5 detected about 2% methylated cfDNA simulant in the non-methylated sample (Fig. 6E). Nearly 100% of methylated cfDNA simulant was detected by Participant Lab 4 and 5 (Fig. 6D, E) in the methylated sample. Participant Lab 1, 2, 3, and 6 were all able to detect methylated cfDNA simulant using an array of different assays (Fig. 6A, B, C, F, G).

## Discussions

Cell free DNA methylation measurements are increasingly utilized for detecting and monitoring diseases such as cancer [39]. The US FDA has approved a few products as companion diagnostic tests, such as Guardant360 Cdx, Epi proColon, and Oncotype DX (www.fda.gov). There are 102 clinical trials (as of December 12th, 2024) using cfDNA methylation as diagnostic and/or monitoring tool for a variety of cancers such as lung, pancreatic, and liver cancer (www.clinicaltrials.gov). These tests and trials highlight the growing importance of cfDNA methylation measurements in clinical diagnostics and personalized medicine. The accuracy of the cfDNA methylation measurements is crucial for the decision-making in a clinical setting. High accuracy of cfDNA methylation measurements ensures that the tests can distinguish between healthy and diseased states, and therefore can be implemented for early-stage cancer detection, relevant therapeutic intervention, sensitive disease state monitoring, and reduce false positives and false negatives [40–42]. Despite the advances in nucleic acid detection technologies in recent years, cfDNA methylation measurements are still challenging [43, 44]. Well-characterized cfDNA methylation reference materials are an unmet need for harmonizing the cfDNA measurements from different labs and platforms and increasing the confidence in cfDNA methylation measurements. In this study we reported candidate cfDNA methylation reference materials that are well-characterized and validated in an interlab study.

The GIAB cell line HG002 genome has previously been extensively characterized [29–31], which makes the assay design and bioinformatics analysis easier when using this particular cell derived genomic DNA as reference materials. Given that the low abundance of cfDNA in human plasma, it is prohibitively difficult and expensive to obtain sufficient biological cfDNA for reference materials. We utilized the well-characterized HG002 genomic DNA to generate the cfDNA simulate, by fragmenting the gDNA and selecting for fragment size distribution relevant to cfDNA. Two formats of candidate cfDNA methylation reference materials were developed independently at NIST and LGC SeraCare from HG002 genomic DNA in this study. NIST candidate cfDNA methylation reference materials included 0%, 5%, 25%, 50%, and 100% methylated cfDNA samples in each set. LGC SeraCare candidate reference materials had non-methylated and methylated cfDNA samples. No significant size distribution bias was found between the methylated and non-methylated cfDNA. NIST unmethylated and methylated samples were found to show similarly reference gene copy numbers (Fig. 1C) and bisulfite conversion recovery rates with different kits (Fig. 1E, F, for Kit A and Kit B respectively). However, LGC SeraCare unmethylated samples were found to have higher reference gene copy numbers per nanogram of cfDNA (Fig. 1D), and the recovery rate from bisulfite conversion reactions was slightly lower than the methylated sample (Fig. 1E, F, for Kit A and Kit B respectively). One of the possible explanations was that there was slight bias generated by the PCR amplification and/or that close to 0% genome-wide methylation makes the material more susceptible to degradation during conversion. The NIST candidate reference materials were found to be homogeneous among the aliquots (Fig. 3) and stable for at least 30 months (Fig. 3F).

The interlab study results showed that the designed percentage of methylation of cfDNA was very well correlated with the output measurements of each lab for the targets with minimum or no baseline methylation (including percent of methylated cfDNA, percent of internal controls) for NIST candidate reference materials (Fig. 5). For the targets with baseline methylation, the additional methylation above baseline level can be calculated by establishing a standard curve using NIST candidate reference materials (Fig. 5B and E). Nearly 100% methylation was detected in LGC methylated samples (Figs. 2 and 6C and D), and no or minimal baseline methylation was detected in the non-methylated sample (Figs. 2 and 6). Participant lab 2 used a methylation-specific assay developed with strict design parameters to maximize specificity. Coupled with harsh bisulfite converted DNA condition and low input DNA leads to variable assay efficiency at the three loci but are consistent within each assay itself across plates (Figs. 5C and 6B.) These results illustrated the utility of the candidate reference materials and supported the commutability of the candidate reference materials. Although the current format of LGC candidate reference materials only includes 0% and 100% methylated samples, these samples can potentially be mixed to create a series of methylated cfDNA percentages. These mixtures could serve as calibrators similar to NIST samples upon validation.

This interlaboratory study demonstrated that the candidate reference materials have significant potential clinical relevance. The validated candidate cfDNA methylation candidate reference materials may help harmonizing cfDNA methylation measurements and ensuring consistency and reliability across different clinical laboratories. Reference materials can enhance the accuracy of diagnostic cfDNA methylation measurements and subsequently lead to better detection of diseases such as cancer or monitoring of disease treatment. The candidate reference materials validated in an interlaboratory study can also ensure that the results are reproducible, which is crucial for clinical applications. The validation of candidate cfDNA methylation reference materials also can support the development and validation of new cfDNA methylation biomarkers, new cfDNA methylation assays, and facilitate their use in clinical trials or even in clinical practice. Taken together, the validated candidate reference materials can provide rigorous quality control measurements for clinical testing and benchmark for evaluating the performance of cfDNA methylation measurements.

This study had several limitations. First, the candidate reference materials were obtained either from fragmented and size selected HG002 genomic DNA or whole genome amplified by PCR, due to the limited amount of cfDNA from real-world samples (plasma, saliva, or urine). The advantage of using Genome-in-a-Bottle HG002 genomic DNA is that the genome sequence has been extensively characterized [29–31]. It may help with the downstream bioinformatics analysis or assay design. The fragmentation profile (position of breaks and size distribution) may be different from the real-world cfDNA profile of clinical samples. Second, this study had a limited number of participant laboratories and limited number of platforms. Third, there were interlaboratory variabilities identified possible due to variations in equipment, protocols, and expertise across the participant labs. Fourth, the cytosine conversion efficiency was not taken into consideration. Although we have tested cytosine conversion kits from different vendors and all can reach nearly 100% conversion rate, conversion rate variations from the participant labs could potentially affect the results. Given the limited amount of cfDNA, literature [45] indicated a high uncertainty in the ddPCR assays, which may account for the variations observed between our experiments. Moreover, the candidate reference materials lack whole genome bisulfite sequencing (WGBS) analysis. Attempted whole genome bisulfite sequencing by commercial vendor did not generate sufficient data for reliable analysis.

In conclusion, we have developed candidate cfDNA methylation reference materials that can be used to benchmark cfDNA methylation measurements and validate methylation detection assays. To the best of our knowledge, this is the first cfDNA methylation candidate reference materials that were characterized and validated in an interlab study.

## Data Availability

All data generated in this study is published in this manuscript. Forty sets of NIST candidate reference materials are deposited at NIH Repository.

## Abbreviations

cfDNA: Cell: Free DNA
NIST: The National Institute of Standards and Technology
ddPCR: Droplet digital PCR
DNMT: DNA methyltransferase
gDNA: Genomic DNA
CCNA1: Cyclin A1
VIM: Vimentin
EVL: Ena/VASP-like
RPL32: Ribosomal protein L32
WIF1: Wnt inhibitory factor 1
HOXA9: Homeobox A1
NPY: Neuropeptide Y
RPP30: Ribonuclease P/MRP Subunit P30
EAC: Esophageal adenocarcinoma
HGD: High-grade dysplasia
GSTP1: Glutathione S-transferase Pi 1
RASSF1A: Ras association domain family member 1
WGS: Whole genome sequencing
MGMT: O-6-Methylguanine-DNA Methyltransferase
BSA: Bovine serum albumin
